# Preoperative irrigation and vacuum sealing drainage with antibiotic-containing drainage fluid of foot and ankle wounds improves outcome of reconstructive skin flap surgery

**DOI:** 10.1186/s13018-019-1418-0

**Published:** 2019-11-20

**Authors:** Xu Gao, Hailei Yin, Jixia Sun

**Affiliations:** 1 Department of Orthopedics, Qingdao University, Qingdao City, 266071 People’s Republic of China; 2 Department of Orthopedics, No.971 Hospital of the People Liberation Army, 22 Min-Jiang Rd, Qingdao, 266071 People’s Republic of China; 3 Department of foot and hand trauma surgery, Qingdao Central Hospital, Qingdao, City, 266071 People’s Republic of China

**Keywords:** Flap reconstruction, Bacterial infection, Inflammatory response

## Abstract

**Objectives:**

By observing the infection and soft tissue defect on the wound surface of the foot and ankle, this paper attempts to explore the effect of preoperative irrigation and vacuum sealing drainage with antibiotic-containing drainage fluid (abPI-VSD) on the bacterial quantity and the local inflammatory response at the flap, and further to provide a basis for applying this technique before a reconstructive skin flap surgery of foot and ankle wounds.

**Methods:**

Seventy-five patients were randomly divided into two groups, and all surgeries were done by one physician. The flap reconstructions were done to 31 cases with the abPI-VSD being used (group A); the flap reconstructions were done to the rest 44 cases after wound cleaning using antibiotic irrigation solution without the use of the abPI-VSD (group B). Quantitative bacteriology was made to group A before and after the use of abPI-VSD; quantitative bacteriology was made to group B before and after wound cleaning. Then, the reconstructive skin flap surgery was done. After the surgeries, the time of local inflammatory response at the flap in both groups were recorded. The measured bacterial quantity was evaluated in logarithm and by *t* test.

**Results:**

The bacterial quantity was 3.2 ± 1.9 × 10^7^ cfu/g in group A before the use of abPI-VSD and 2.3 ± 2.0 × 10^7^ in group B (*P* > 0.05) before debridement. The bacterial quantity was 1.2 ± 2.0 × 10^4^ cfu/g in group A after abPI-VSD and was 2.9 ± 4.0 × 10^6^ in group B after wound cleaning (*P* < 0.05). The time of postoperative inflammatory response in the flap was 8 ± 2.5 days in group A and 13 ± 3.4 days in group B (*P* < 0.05).

**Conclusions:**

abPI-VSD can distinctly reduce the bacterial quantity on the surface of the wound, provide a good condition of tissue bed for the flap reconstruction, and effectively control the local inflammatory response at the flap and hence improve the survival quality of the flap.

## Introduction

Severe open foot or ankle injuries (open fractures) are associated with soft tissue defects that in some cases entail bone and tendon exposure. The soft tissue defects are commonly repaired by reconstructive skin flap surgery [1–3]. However, a considerable risk factor in reconstructive surgery is free or pedicle flap infection [4–6], which can reduce the quality of the flap or even lead to necrosis in part of the flap or in the entire graft [7, 8]. Consequently, ensuring that the wound contains minimal bacterial load is key to proper primary wound closure and optimal skin flap transplantation outcome [9, 10].

Several strategies have been employed to reduce or eliminate infections in physical trauma-induced open fractures. These include standard methods such as debridement and occlusive dressing [11, 12] as well as more novel approaches such as dermal regeneration matrices [13] and preoperative irrigation and vacuum sealing drainage [PI-VSD] [8, 14–17], both of which may be accompanied by antibiotic prophylaxis, the application of antibiotic agents [10, 12, 14, 18], photodynamic therapy [19, 20], and the use of wound dressings that reduce biofilm formation [21]. PI-VSD comprises irrigation of the wound followed by the placement of a drainage container over the open wound and the passage of sterile fluid under conditions of negative pressure. As a result, wound exudate, necrotic tissue fragments, and pus are effectively removed and the wound area is kept moist so as to emulate physiological conditions to promote healing and reduce healing time [16]. Rapid elimination of infections and infection-prone material from the wound is critical in expediting primary wound closure by reconstructive surgery [18], which can be effectively achieved by PI-VSD. The modality also increases blood flow to the wound [22] and promotes the accumulation of leukocytes and fibroblasts in the wound that facilitate the healing process [23]. Moreover, PI-VSD reduces edema and third space fluids, the frequency at which wound dressings require changing (and hence the workload of medical staff), and overall medical costs [15, 16, 24]. Accordingly, PI-VSD is increasingly being integrated into reconstructive surgery protocols for open fractures.

Of the abovementioned techniques, PI-VSD was shown to yield better results in open fracture patients than debridement and occlusive dressings. In a recent retrospective study, two cohorts of patients with Gustilo type IIIB open tibial fractures were compared with respect to occlusive dressing versus vacuum sealing drainage (VSD) [25]. In the VSD group, only 10% of the patients exhibited flap failure and 6% had an infection, whereas these statistics were 33% and 11%, respectively, in the occlusive dressing group. Moreover, dermal regeneration matrices such as Medskin Solutions’ MatriDerm and Johnson & Johnson’s Integra are associated with a failure rate of up to 25% [13] and do not address the infection. Although PI-VSD is also associated with occasional mild drawbacks [23], the modality is safe [26] and is of adjuvant value for wound healing [23] and the outcome of reconstructive surgery [15].

The above referenced studies and others have demonstrated that PI-VSD can reduce the degree of infection [27–29], but no studies have examined the effect of PI-VSD with antibiotic-containing drainage fluid (*ab*PI-VSD) on bacterial load and flap quality. Our work therefore focused on the effect of PI-VSD with gentamicin on the bacterial load in physical trauma-induced open foot and/or ankle wounds and the inflammatory response in the graft following reconstructive skin flap surgery. It was hypothesized that *ab*PI-VSD would reduce bacterial load in the pre-surgical wound, reduce inflammation in the post-surgical flap, and improve flap quality. The main findings of the study were that *ab*PI-VSD significantly reduced the incidence of secondary infections on the surface of open fracture wounds, augmented the post-operative quality of the transplanted tissue, and improved the surgical outcome. An optimized protocol for *ab*PI-VSD was formulated.

## Materials and methods

### Patients and clinical presentation

The study was approved by the institutional review board of the No. 971 Hospital of the People Liberation Army and performed in accordance with the Declaration of Helsinki. Written informed consent was obtained from all patients prior to inclusion.

Seventy-five patients who were admitted at the Department of No. 971 Hospital of the People Liberation Army during the period April 2013 to January 2018 were included in the study. Hospitalized patients who presented with soft tissue defects at the foot and ankle, wound infection, and necrotic tissue on the wound surface were included in the study. Where necessary, the patients received upon admission to correct the water and electrolyte disbalance and hypoproteinemia and to improve nutritional status.

### Patient demographics, injury characteristics, and study design

The patient demographics and injury characteristics are presented in Table 1. The patients were divided into group A (abPI-VSD before reconstructive surgery) and group B (wound cleaning using antibiotic irrigation solution before reconstructive surgery). The reconstructions in both groups were performed with a pedicle flap.

**Table 1:** Patient demographics and injury characteristics

	Group A	Group B
Patients [75]	31	44
Male [52]	21 (28%)	31 (41%)
Female [23]	10 (13%)	13(18%)
Mean ± SD age [years]	37.7 ± 7.3	36.5 ± 5.6
Size of the injury [cm^2^]	19.4 ± 5.3	21.1 ± 6.2
Mean ± SD time between injury and surgery [days]	15.8 ± 4.3	17.4 ± 6.7
Type of injury [75]		
Traffic accident [39]	17 (23%)	22 (30%)
Crushing injuries from stones or machines [23]	11 (15%)	12 (16%)
Machine squeezing or twisting [10]	2 (2%)	8 (11%)
Metallurgical hot press [3]	1 (1%)	2 (2%)

### Wound care, preoperative irrigation, and vacuum sealing drainage

**Fig. 1:**
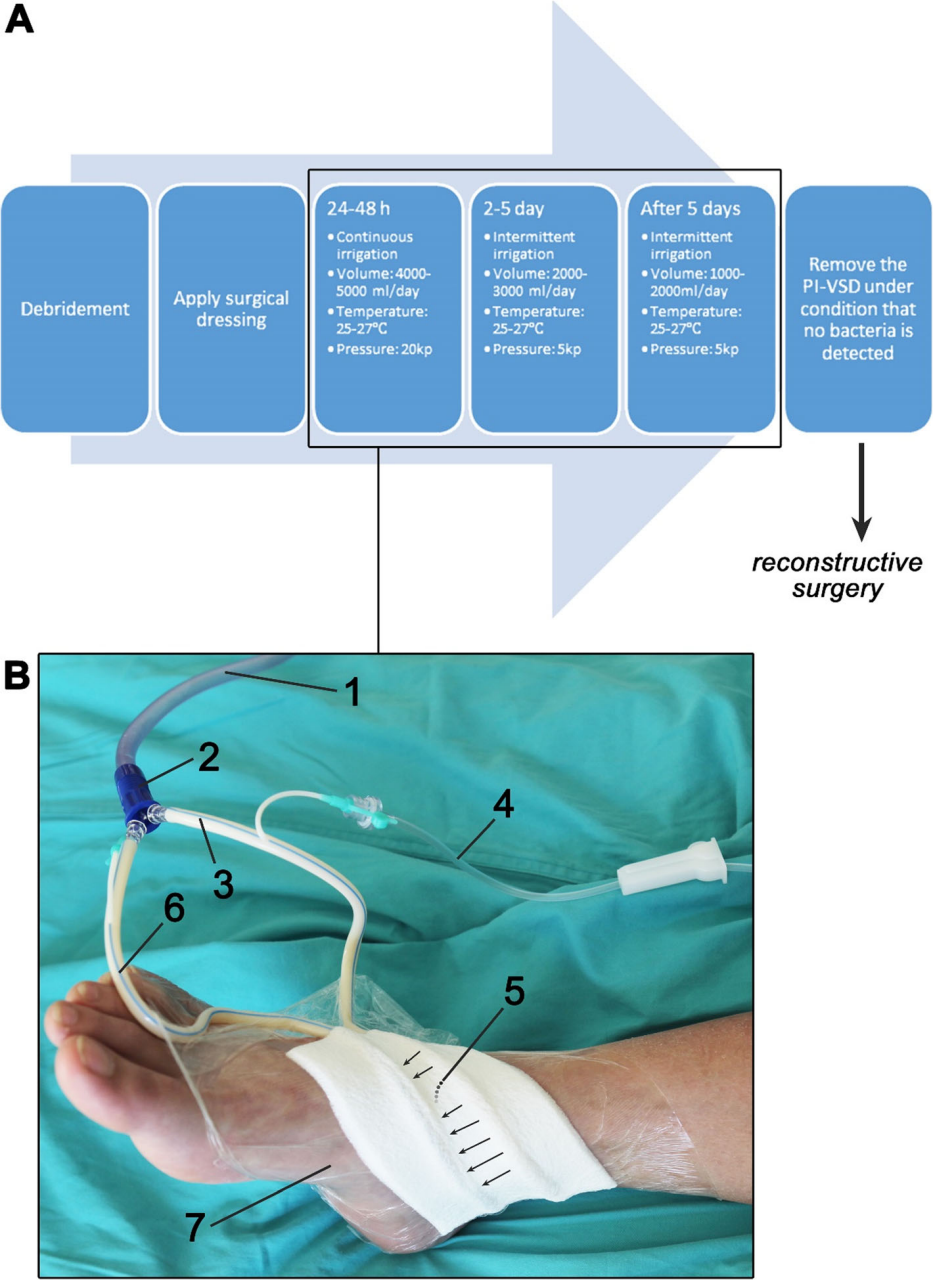
**a** Summary of the *ab*PI-VSD procedure. **b** Image of *ab*PI-VSD implemented on a patient with traffic accident. The shown components of the *ab*PI-VSD system include (1) main tube connected to the medical suction unit and a drainage container, (2) two-way connector, (3) main drainage pipe, (4) gentamycin-containing irrigation solution supply, (5) multi-hole foam cushion that is placed over the wound through which the drainage fluid is passed (arrows indicate direction), (6) tube that drains the fluid, and (7) breathable film for adhesion and sealing

The *ab*PI-VSD procedure is summarized and depicted in Fig. 1. In group A, the wound was cleaned with gentamycin-containing irrigation solution (160,000 U gentamicin sulfate (Chenxin Pharm, Shandong, China) in 500 mL of sterile 0.9% sodium chloride solution (Siyao, Shijiazhuang, China)) and the necrotic tissue was trimmed. The multi-hole foam cushion was trimmed in conformity with the shape of the wound and fixed to the wound with medical film (Fig. 1b). Next, the drainage bottle (Beijing Xinghua Instrument Company, Beijing, China) and the negative pressure pump (ZN100, Yantai Jianyuan Tech, Yantai, China) were connected.

Continuous irrigation was applied during the first 24–48 h at a volume of 4000–5000 mL/day. The solution was kept at 25–27 °C with a temperature controller (Sanyo, Osaka, Japan), and the irrigation was performed at a negative pressure of 20 kPa to avoid clogging of the drainage tube. After 2–3 days, when the amount of floccules in the drainage liquid had receded and the color had faded, irrigation was performed intermittently (3 times/day) at 2000–3000 mL/day and a negative pressure of 5 kPa. After 3–5 days, when the drained liquid had cleared up, the volume was further lowered to 1000–2000 mL/day. Intermittent irrigation (2 times/day) was continued for 3–5 days. After the drained liquid was free of bacteria (assessed by bacterial culture at the Department of Clinical Microbiology, NO. 971 Hospital of PLA), the PI-VSD was removed.

In group B, the wound was cleaned by debridement using gentamycin irrigation solution. Then, the skin flap transplantation was performed.

### Determination of bacterial load

In group A, the bacterial load was determined before and after *ab*PI-VSD, whereas in group B, the bacterial load was determined before and after debridement. Seventy-five percent of alcohol was used to disinfect the wound surface twice. Two parallel incisions were made in the center of the disinfection area, 1 cm in length and 1 cm in width, and a tissue biopsy (9–55 mg) was acquired from the wound surface center and weighed. The specimen was suspended in sterile saline solution at a 1:99 weight to volume ratio and homogenized in a glass homogenizer. The homogenate was serially diluted 10× with sterile saline solution (Siyao). A 10-cm diameter agarose plate was inoculated with the 100 μL-fold dilution and incubated at 37 °C for 24 h. The number of bacterial colonies was counted under a microscope. The bacterial load in each wound was calculated as the number of bacterial colonies × 10 × dilution factor.

### Pedicle flap reconstructive surgery

The reconstructive skin flap surgery was performed by one physician. All the wound surfaces were reconstructed using an antegrade and retrograde pedicle flap, local transplantation, subcutaneous tunnel, and cross leg flap.

### Quantification of the extent of graft inflammation

After surgery, the local inflammatory response in and around the flap was monitored. Inflammation was determined by the time it took for the flap temperature to drop. The extent of inflammation in the pedicle flap was determined by the C-reactive protein (CRP) levels.

### Statistical analysis

The bacterial number in each gram of tissue ± standard deviation cfu/g means that by using the statistical software SPSS (SPSS, Chicago, IL), take the logarithm of the bacterial number and conduct the independent-samples *t* test; *P* ≤ 0.05 is of statistical significance. The flap local inflammatory response time is measured by the independent-samples *t* test.

## Results

There were no statistical differences between the two groups with respect to gender, age, area of injury, and time frame between injury and surgery (*P* > 0.05).

**Fig. 2:**
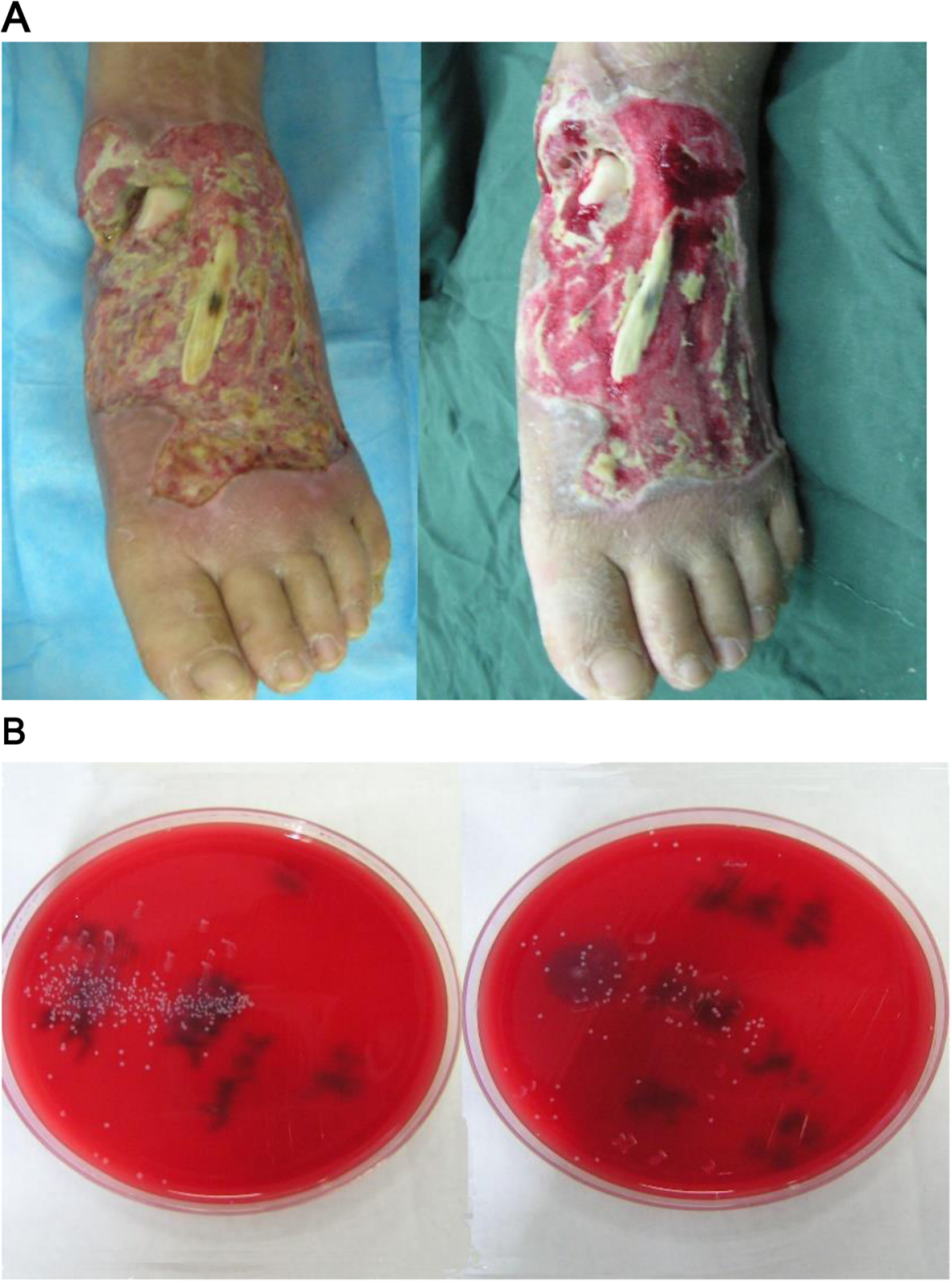
**a** Wound changes before and after PI-VSD application. Squeezing the soft tissue defect, the wound is red and swelling and has a smell (left side). After radical debridement through PI-VSD for 1 week, the wound is clean, the granulation tissue is fresh, and the infection is controlled (right side). **b** Quantitative bacterial before and after PI-VSD application. The quantitative bacterial before and after PI-VSD application is 0.9 × 10^7^ cfu/g (10,000×) (left side) and 6.5 × 10^4^ cfu/g (100×) (right side). The bacterial quantity is obviously reduced

The necrotic spots as well as the red appearance and swollen nature of the wound were obvious. The wounds exhibited extensive purulent secretion and a particular smell. After abPI-VSD treatment for 5–7 days, the granulation tissue at the wound surface grew fast and was fresh and red, and the exudation at the wound surface was distinctly reduced and the infection was also controlled (Fig. 2).

### The comparison of bacterial quantity between groups A and B (Table 2)

The bacterial quantity was 3.2 ± 1.9 × 10^7^ cfu/g in group A before the use of abPI-VSD and 2.3 ± 2.0 × 10^7^ in group B (*P* > 0.05) before debridement. So far, the acknowledged bacterial infection valuation standard is: when the bacterial number excels 10^5^ per gram of tissue or per milliliter of liquid, it is considered to be an infection. Therefore, both groups A and B are infected wounds.

**Table 2:** Bacterial quantity and flap local inflammatory reaction time

Group	*N*	Before using PI-VSD, bacterial quantity	Pre-reconstructive surgery, bacterial quantity	Flap local inflammatory, reaction time [days]
A	31	3.2 ± 1.9 × 10^7 △^	1.2 ± 2.0 × 10^4^**	8 ± 2.5**
B	44	2.3 ± 2.0 × 10^7△^	2.9 ± 4.0 × 10^6^**	13 ± 3.4**

Bacterial quantity unit: cfu/g

**Comparison between the two groups, *P* < 0.05

^△^Comparison between the two groups, *P* > 0.05

The bacterial quantity was 1.2 ± 2.0 × 10^4^ cfu/g in group A after the use of abPI-VSD procedure and was 2.9 ± 4.0 × 10^6^(*P* < 0.05) in group B after debridement using gentamycin irrigation solution.

### Postoperative flap local inflammatory reaction time (Table 2)

After the surgery, time of flap local inflammatory response was observed and recorded, which is 8 ± 2.5 days in group A and 13 ± 3.4 days in group B (*P* < 0.05).

## Discussion

Between April 2007 and January 2009, our hospital treated 75 patients with a foot and/or ankle infection and soft tissue defects. abPI-VSD technique was adopted before all the surgeries to control bacterial growth and clean the wound. During the surgery, different pedicle flap coverage was chosen according to the location and size of the tissue defect. The wound was cured and the local blood supply was improved in one stage, and the patients recovered well from the foot and ankle infection, which displays a rather satisfactory effect.

### Influence of wound surface bacterial quantity on the flap

Chang and Mathes [30] observed that the intracutaneous inoculation of *Staphylococcus aureus* to dog flap could cause flap necrosis. Pearl [31] found that compared to any other types of flaps, axial flaps and myocutaneous flaps have higher ability to eliminate the inoculated bacteria under flaps and that any type of flap, after treated by a small dose of phenoxybenzamine to increase its blood supply, has an obviously higher ability to eliminate bacteria, which demonstrates that the ability of myocutaneous flaps to eliminate bacteria is determined by blood supply.

Compared with any other types of flaps, myocutaneous flaps are abundant in blood supply and have higher ability to eliminate wound surface bacterial when the wound surface is considered to be moderate pollution (10^5^/g tissue). However, myocutaneous flaps look bloated and swollen and damage the donor greatly, which limits its application in clinical practice. Most importantly, if the bacterial quantity is too large, myocutaneous flaps get infected easily and influence the survival quality of flaps, and even worse, cause flap necrosis or partial necrosis. Therefore, before reconstructing infection and soft tissue wound surface on the foot and ankle, measures such as local application of sensitive antibiotics or PI-VSD should be used to control bacterial growth.

### Influence of PI-VSD application on wound surface bacterial quantity

In recent decades, with the theory of wound “moist healing” [32] being put forward, numerous studies have proven that PI-VSD technique can provide a moist environment for foot and ankle wound, helping the wound to heal and, in the meantime, reducing the bacterial quantity and alleviating the infection on the wound surface.

Weed et al. [33] found in animal studies that the use of VAC can decrease the bacterial quantity. Polykandriotis et al. [34] applied PI-VS D to 9 cases of hand injury and effectively cured the hand defects and prevented them from getting infected. PI-VSD turns open wound to closure wound, preventing the invasion of bacteria, and forms a slow liquid flow from the wound to the dressing, making the vital cells to be kept and wastes such as the flaccid cells and non-nutritious interstitial fluid to be suctioned and reducing the culture medium for bacterial survival and growth.

PI-VSD can reduce the foot and ankle wound surface infection, which might be related to the fact that a closure and moist environment is better for the immune cells in the organism to function [35]. Compared to the dry wound surface formed under the traditional mullauflage, the moist wound surface formed under the enclosed dressing is better for the polymorphic nucleus eukopenia to infiltrate, which offers a good local environment for the immune cells to phagocytose and eliminate bacteria, and further reduces the bacterial quantity. We assume that vacuum sealing drainage therapy is in a position to become a reliable sealing technique for temporary soft tissue defect and bone exposure. The result of this experiment showed that vacuum aspiration effectively eliminate tissue degradation product and pollutant, decrease bacterial quantity, control infection, and improve the survival quantity of the flap.

### Preoperative PI-VSD reduces damage to the donor and receptor site during surgery

For the infected wound surface on the foot and ankle, to apply PI-VSD before the surgery can not only clean the exudates and reduce the local tissue edema, but also promote the production of granulation tissue, speed the healing and epithelialization of the wound, and play a role in slow serial debridement. Clinical observation shows that after the use of PI-VSD and slow debridement of the wound surface, the granulation tissue is healthy and fresh (Fig. 2). Therefore, in regard to seriously infected wound surface, adopting preoperative PI-VSD and the relatively conservative serial debridement instead of radical debridement, and then doing the flap reconstruction, can retain important tissue as much as possible. On the premise of using PI-VSD to reduce the bacterial quantity on the wound surface, carrying on serial debridement decreases the damage to important structure in the donor during the surgery.

Musculocutaneous flaps and fasciocutaneous flaps used to cover the wound surface of ankle joint soft tissue can be divided into many types, including arteria peronea retrograde flap, medial crural flap, sural neurotrophy vessel flap, saphenous neurotrophy vessel flap, and cross-leg flap. The choice of flaps is determined by the conditions of ankle joint, pretibial and posterior tibial artery, and the soft tissue on the affected side and around the ankle joint. If there is soft tissue edema around the ankle joint, or the soft tissue or the artery is in a bad condition, the cross-leg flap with better tissue can be chosen for the reconstruction. For patients who need flap retrograding island transposition of sural neurotrophy vessel and saphenous neurotrophy vessel, the acerated ends of the sural nerve or the saphenous nerve on the flap lateral margin can be anastomosed with the medial plantar or lateral plantar nerve.

To merge an open infection on the ankle joint, according to the position of the focus and the condition of the infected side body, choose different musculocutaneous flap and fasciocutaneous flap to cover the wound, and at the same time renew the impermeability of the ankle joint cavity. According to the postoperative drug sensitive test, use local antibiotic solution perfusion and constant vacuum sealing drainage through tube to treat the joint infection, or use ozonated water or silver nitrate solution [30] to eliminate the bacterial.

**Fig. 3:**
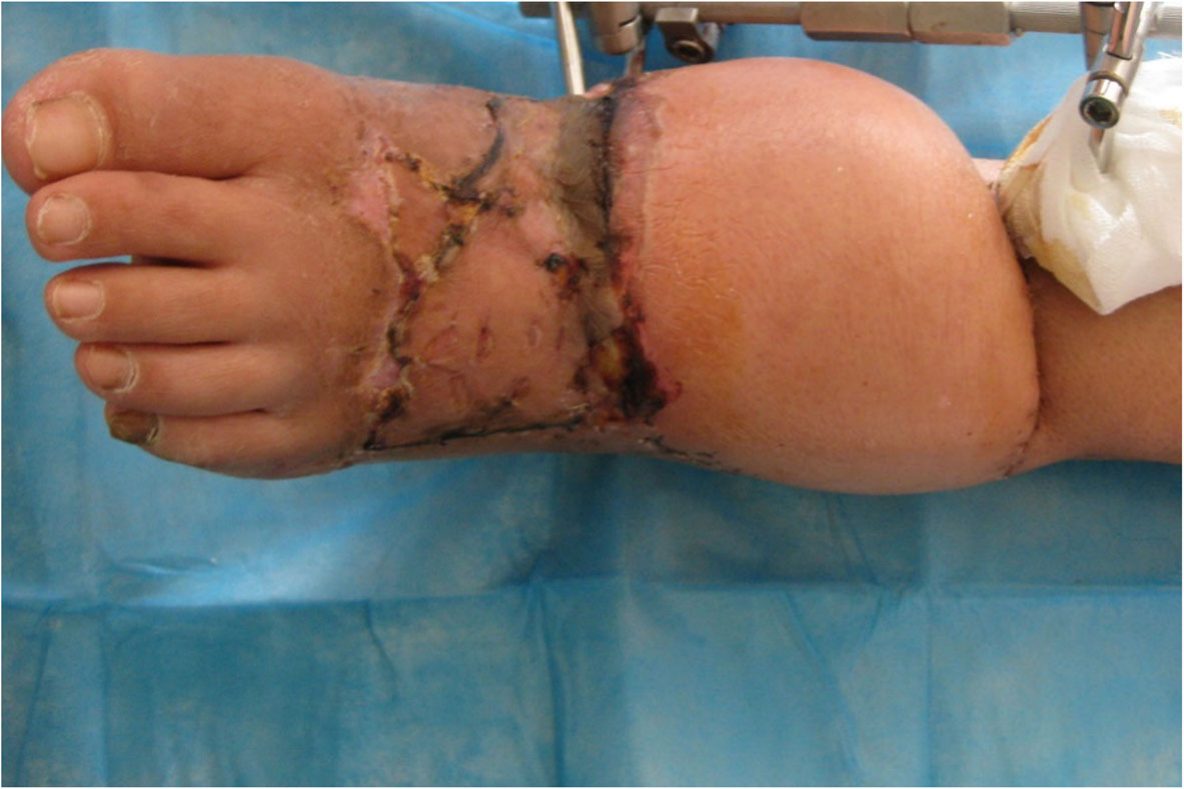
After flap surgery, the flap is good, red and moist, not swelling, without exudation and with normal temperature. The granulation on the foot dorsal front side is fresh. Medium thickness flap is used to do the skin graft; the skin graft survives and is in good condition

For large scale skin tissue defect, adopting medium thickness skin graft under vacuum aspiration on the healthy granulation before and under the surgery can obviously reduce the defect scale of skin soft tissue (Fig. 3). However, for the reconstruction of functional location (such as heel, the first and fifth metatarsal) and bone and tendon exposure, the medium thickness flap cannot be used, and usually, the relatively smaller flap can be used to reduce effectively the damage to the donor caused by the oversized flap.

The results of the experiment show that vacuum aspiration eliminate tissue degradation product and pollutant and control bacterial infection. The bacterial quantity was 3.2 ± 1.9 × 10^7^ cfu/g in group A before the use of abPI-VSD and was 2.3 ± 2.0 × 10^7^ cfu/g (*P* > 0.05) in group B before debridement, which illustrate that the degree of wound surface pollution in the two groups are the same. Group A used treatment measure to control bacterial growth for 5–7 days, and the bacterial quantity on wound surface reduces from 3.2 ± 1.9 × 10^7^ to 1.2 ± 2.0 × 10^4^ cfu/g (*P* < 0.05). The operative inflammatory response time was 8 ± 2.5 days in group A and 13 ± 3.4 days in group B (*P* > 0.05).

## Conclusion

The experiment proves that the application of abPI-VSD before the surgery for foot and ankle infection and soft tissue defect can reduce the damage to the donor and the receptor site in the surgery, decrease distinctly the bacterial quantity on wound surface, and further reduce the local inflammatory response at the flap, which are helpful to the flap quality. PI-VSD technique with antibiotic-containing drainage fluid has great value in clinical application.

## Data Availability

Not applicable

## Abbreviations

abPI-VSD: Preoperative irrigation and vacuum sealing drainage with antibiotic-containing drainage fluid
PI-VSD: Preoperative irrigation and vacuum sealing drainage
VSD: Vacuum sealing drainage
CRP: C-reactive protein
